# Efficacy of HEPA Air Cleaner on Improving Indoor Particulate Matter 2.5 Concentration

**DOI:** 10.3390/ijerph191811517

**Published:** 2022-09-13

**Authors:** Chiu-Fan Chen, Chun-Hsiang Hsu, Yu-Jung Chang, Chao-Hsien Lee, David Lin Lee

**Affiliations:** 1 Division of Chest Medicine, Kaohsiung Veterans General Hospital, Kaohsiung 813, Taiwan; 2 Kaohsiung and Pingtung Branch, National Health Insurance Administration, Ministry of Health and Welfare, Kaohsiung 801, Taiwan; 3 Department of Nursing, Meiho University, Pingtung 912, Taiwan; 4 Department of Medicine, National Yang-Ming University, Taipei 112, Taiwan

**Keywords:** air cleaner, air pollution, HEPA, PM_2.5_

## Abstract

High-efficiency particulate air (HEPA) filters is a potential tool used to remove fine particles and improve indoor air quality. This study aims to analyze the real-world efficacy of portable HEPA air cleaners in a household environment. Laser light dispersion PM_2.5_ sensors are used to continuously monitor the indoor and outdoor PM_2.5_ level before and after HEPA air cleaner filtration. Overall, HEPA air cleaners significantly reduce the indoor PM_2.5_ level (33.5 ± 10.3 vs. 17.2 ± 10.7 µg/m^3^, mean difference (MD) = −16.3 µg/m^3^, *p* < 0.001) and indoor/outdoor PM_2.5_% (76.3 ± 16.8 vs. 38.6 ± 19.8%, MD = −37.7%, *p* < 0.001). The efficacy to reduce PM_2.5_ is strongest in three machines with medium-flow setting group (indoor PM_2.5_ MD: −26.5 µg/m^3^, indoor/outdoor PM_2.5_ percentage MD: −56.4%). Multiple linear regression demonstrates that outdoor PM_2.5_, machine number, airflow speed, and window ventilation are significant factors associated with indoor PM_2.5_ concentrations (R = 0.879) and percentage of the indoor/outdoor PM_2.5_ ratio (R = 0.808). HEPA air cleaners can effectively improve indoor PM_2.5_ air pollution. Adequate air cleaner machine numbers, appropriate airflow, and window ventilation limitations are important to achieve the best efficacy of the HEPA air cleaner.

## 1. Introduction

Air pollution is one of the most important global health issues. It has already become the 4th leading risk factor of early death worldwide in 2019, and accounts for 6.67 million deaths from all causes [1,2]. Particulate matter 2.5 (PM_2.5_), defined as small particles of aerodynamic diameter <2.5 µm, represents the major air pollutants in both outdoor and indoor environments. During respiration, PM_2.5_ can reach deeply into the alveoli and enter the blood circulation, resulting in nearly all kinds of organ system damage by inducing local and systemic inflammation via oxidative stress, immune and inflammation dysregulation, and altered gene expression [3,4]. The current literature has shown a clear association between PM_2.5_ exposure and the development of lung cancer, chronic obstructive pulmonary disease (COPD), lung function decline, asthma, and pneumonia [5,6,7,8,9]. Besides, PM_2.5_ also contributes to ischemic heart disease and cerebrovascular disease [10].

The global mean PM_2.5_ in 2019 was 43 µg/m^3^ [1,2]. The highest mean PM_2.5_ level has been reported in East and South Asia (India 83.2 µg/m^3^, Nepal 83.1 µg/m^3^), the Middle East (Saudi Arabia 62 µg/m^3^), and Africa (Egypt 67.9 µg/m^3^), whereas the lowest PM_2.5_ level was in the United States (7.7 µg/m^3^), United Kingdom (10 µg/m^3^), Canada (7.1 µg/m^3^), and Australia (6.7 µg/m^3^) [2]. In Taiwan, the mean PM_2.5_ data was 23 µg/m^3^ [2]. The PM_2.5_ data also varies among different seasons in Taiwan, with the highest reported during winter and the lowest during summer [11,12]. In Kaohsiung, a city in Southern Taiwan, the average PM_2.5_ data may reach up to 50 µg/m^3^ during the winter season [11,12]. Although the yearly mean PM_2.5_ level has gradually improved over the past 15 years in Taiwan, air pollution caused by PM_2.5_ is still severe in middle and southern Taiwan [12]. PM_2.5_ can easily infiltrate buildings through the windows. Literature has shown that people spend an average of 87% of their time indoors [13]. Therefore, indoor air quality control is an important and effective method to reduce PM_2.5_ exposure.

The high-efficiency particulate air (HEPA) filter is a type of fibrous media air filter that effectively removes ≥ 99.97% of 0.3-µm fine particles from the air [14]. Theoretically, a portable HEPA filter is an ideal and effective method to reduce the indoor PM_2.5_ level and improve air quality. However, previous studies have demonstrated variable efficacy of portable HEPA air cleaners on reducing indoor PM_2.5_ (29–66%) [15,16,17,18,19,20]. This considerable variation is a warning that HEPA air cleaner efficacy could be severely impaired by certain factors. It has been reported that higher window opening frequency is associated with worse air cleaner efficacy [15,20]. Air cleaner use time (in other words: compliance) is also an obvious problem affecting air cleaner efficacy [20]. Air cleaner use pattern and HEPA filter condition (new vs. old) are other factors that require further study [15,18]. Therefore, in this study, we aim to evaluate the real-world efficacy of portable HEPA air cleaners and to analyze its association with the possible factors.

## 2. Materials and Methods

**Figure 1:**
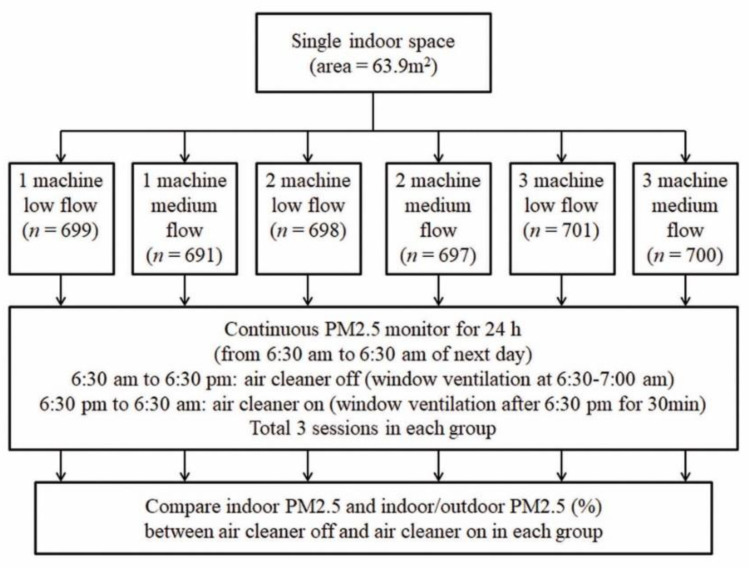
Flow chart of air cleaner study. For each experiment (24 h each time, totaling 3 times in each group), in the first 12 h the air cleaner is turned off. Then in the following 12 h, the air cleaner is turned on to evaluate the efficacy of indoor PM_2.5_ removal.

In this study, portable HEPA air cleaners (F-VXH50W, Panasonic, Japan) are tested and laser light dispersion PM sensors are used to continuously monitor PM_2.5_ level. This study was conducted in a single indoor space in Kaohsiung City, Taiwan, from October 2020 to April 2021. A graphic of the air cleaner study is shown in Figure 1. Six groups of air cleaner experiments were designed according to different machine numbers (1, 2 or 3) and airflow setting (low flow and medium flow) (Figure 1). In each group, the experiments were performed over 3 separate days (24 h each time, total 72 h). The indoor and outdoor PM_2.5_ levels were measured continuously, starting from 6:30 a.m. to 6:30 a.m. the next day. In the first 12 h (6:30 a.m.–6:30 p.m.), the air cleaner was turned off (Control group). In the following 12 h (6:30 p.m.–6:30 a.m. of the next day), the air cleaner was turned on to evaluate its efficacy on indoor PM_2.5_ level (Intervention group).

The PM_2.5_ level was measured using an AirBox (AI-1001W V2 and AI-1001W V3, Edimax, Taipei, Taiwan) that is equipped with Plantower PMS5003, laser light dispersion technique-based PM sensor. The AirBox continuously measures the PM_2.5_ levels at 6-min interval. The accuracy of this PM_2.5_ sensor has been validated previously and showed good correlation (regression coefficient R = 0.82–0.99) compared with professional PM monitors [21,22,23]. A correlation test was performed to confirm the reliability and accordance of the two PM_2.5_ sensors used in our study and showed a very good correlation (R = 0.99, R^2^ = 0.98, Figure S2).

The study environment was an apartment in Kaohsiung City, Taiwan, with an indoor area of 63.9 m^2^ and a height of 280 cm. The floor plan of the apartment shows the positions of two AirBox PM_2.5_ sensors and three air cleaners (Figure S3). The AirBox was implanted in the middle of the apartment to measure the indoor PM_2.5_ level, and another AirBox was implanted on the balcony to measure the outdoor PM_2.5_ level. A window-open protocol was developed to standardize the window ventilation condition during the study. Window ventilation period: the large window of the living room (102 × 192 cm) was only opened 30 min twice a day (6:30–7:00 a.m., and then at night after 6:30 p.m.). The small windows of the kitchen (60 × 57 cm) and two bathrooms (25 × 43 cm, 25 × 80 cm), which are located on the same side of the apartment, were kept open all day to maintain minimal ventilation. Serial CO_2_ was measured using a portable CO_2_ monitor (GC-2028, Lutron Electronics Inc., Coopersburg, PA, USA) to evaluate the safety and effectiveness of this window-open protocol (Figure S1). The results show marginal CO_2_ elevation at the end of the test (mean 1014 ppm, range: 841–1208 ppm), slightly above the recommended upper normal limit for indoor CO_2_ (1000 ppm) [24].

The indoor PM_2.5_, outdoor PM_2.5_, and indoor/outdoor PM_2.5_ percentage were collected. The outdoor wind speed data was collected from the Environmental Protection Administration of Taiwan. The features of the air cleaner were also evaluated, including the airflow speed, noise, air outlet size, and size of the HEPA filter. The airflow speed was measured in the middle of the airflow outlet using a portable airflow meter (LM-81 AM, Lutron Electronics Inc., Coopersburg, PA, USA). The air cleaner noise was measured at a distance of 1 m from the airflow outlet using a portable noise meter (TM-102, TENMARS Electronics Co. Ltd., Taipei, Taiwan). Air filtration volume was calculated to estimate the clean air delivery rate (CADR) at each airflow speed setting.

The primary outcome is indoor/outdoor PM_2.5_ percentage. The secondary outcomes are indoor and outdoor PM_2.5_ level, mean change in indoor PM_2.5_, and mean change in indoor/outdoor PM_2.5_ percentage after air cleaner filtration. Factors associated with indoor PM_2.5_ and indoor/outdoor PM_2.5_ percentages are also secondary outcomes.

Continuous variables are expressed as mean ± standard deviation. Independent *t*-tests were used for two-group comparison, whereas one-way analysis of variance (ANOVA) was used for multiple-group comparisons. Categorical variables are expressed as numbers (percentage) and were compared using the chi-square test or Fisher’s exact test if the expected count in any cell was < 5. Multiple linear regression was used to analyze the factors associated with PM_2.5_ outcomes. Statistical analysis was performed using IBM SPSS Statistics for Windows, Version 20.0 (IBM Corp., Armonk, NY, USA). The differences were considered significant at a two-tailed *p*-value of < 0.05.

## 3. Results

The features of HEPA air cleaners tested in this study are shown in Table 1. The airflow speed and estimated CADR of the medium-flow setting are approximately three-fold of the low-flow setting. The three-fold flow difference is also observed between the medium-flow and the high-flow setting. The high-flow setting showed much louder noise production, which is intolerable when continuously used. The HEPA filter size was 40.5 × 24.5 × 3.5 cm. During the initial 12 h period of experiment when the air cleaner was s turned off, the indoor and outdoor PM_2.5_ levels were highly correlated. The average indoor/outdoor PM_2.5_ ratio is 0.76 ± 0.17, and the linear regression shows R = 0.794.

**Figure 2:**
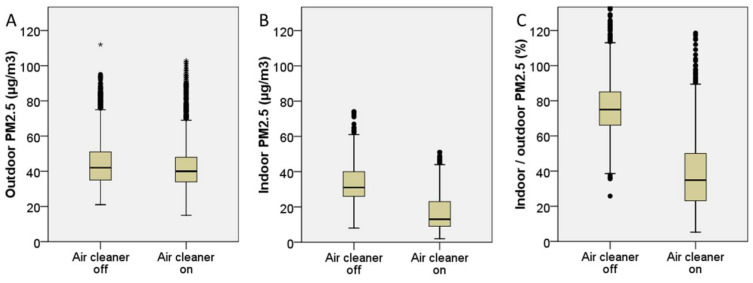
Overall changes in indoor and outdoor PM_2.5_ levels before and after air cleaner use: (**A**) outdoor PM_2.5_; (**B**) indoor PM_2.5_; and (**C**) indoor/outdoor PM_2.5_ percentage. * outliers.

**Figure 3:**
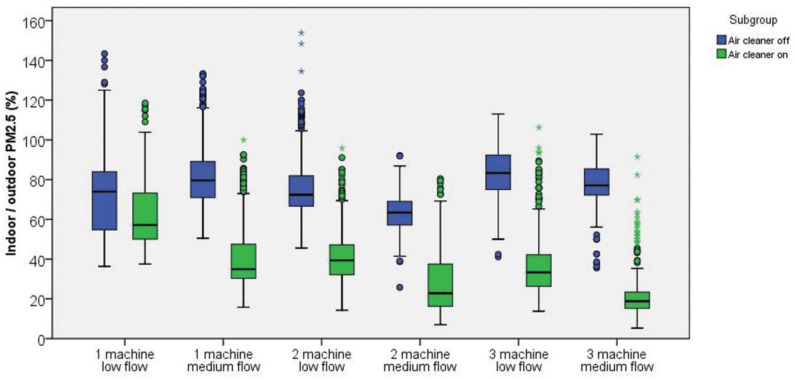
Details of the changes in indoor/outdoor PM_2.5_ percentage of each study group before and after air cleaner use. Remarkable improvements of indoor/outdoor PM_2.5_ percentage are noted after air cleaner use, except for the 1 machine low flow group. * outliers.

**Figure 4:**
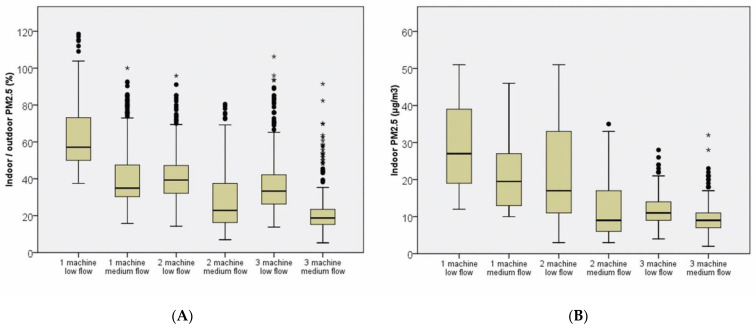
Box plots of PM_2.5_ outcomes in the six groups: (**A**) indoor/outdoor PM_2.5_ percentage and (**B**) indoor PM_2.5_ levels. More air cleaner machines and higher flow speeds are significantly associated with better indoor PM_2.5_ level. * outliers.

The overall indoor and outdoor PM_2.5_ levels before and after air cleaner filtration are shown in Figure 2 and Table 2. Significant improvement is observed in the indoor PM_2.5_ level (33.5 ± 10.3 vs. 17.2 ± 10.7 µg/m^3^, mean difference (MD) = −16.3 µg/m^3^, *p* < 0.001) and indoor/outdoor PM_2.5_ percentage (76.3 ± 16.8 vs. 38.6 ± 19.8%, MD = −37.7%, *p* < 0.001) after the start of HEPA air cleaners. By contrast, no significant change is observed in the outdoor PM_2.5_ level (44.7 ± 13.8 vs. 44.6 ± 16.8, MD = −0.1 µg/m^3^, *p* = 0.875). The detailed PM_2.5_ data of each study group are shown in Table 2, Figure 3 and Figure 4 and Figure S4. All six groups of HEPA air cleaners show significant improvement in indoor PM_2.5_ level after air cleaner use (MD range: −3.7 to −26.5 µg/m^3^) and indoor/outdoor PM_2.5_ percentage (MD range: −10.4% to −56.4%). The detailed trends of indoor/outdoor PM_2.5_ percentage in the six study groups are shown in Figure S5. The distributions of outdoor wind speed in each study group are shown in Figure S6, and a one-way ANOVA shows a significant difference between the groups (*p* < 0.001).

**Figure 5:**
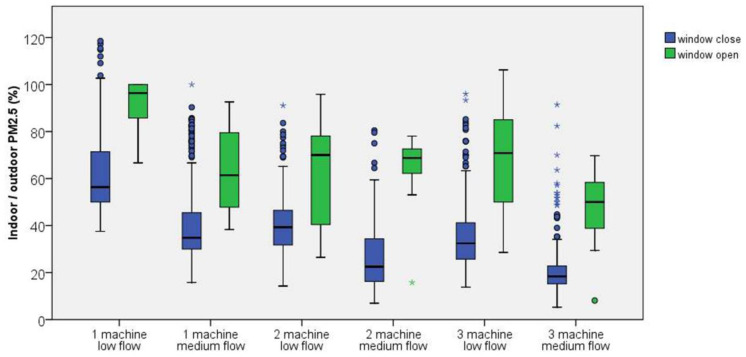
Details on the influence of living room window ventilation on the efficacy of air cleaners in each study group. The efficacy of HEPA filter air cleaner is severely impaired during period of window open for ventilation. * outliers.

The influence of living room window ventilation on the efficacy of the air cleaner is shown in Figure 5 and Table 3. Overall, indoor/outdoor PM_2.5_ percentage is significantly higher during the window open period than that during the window close period (65.3 ± 21.5 vs. 37.5 ± 18.9%, MD = 27.8%, *p* < 0.001). A significant difference is found in all six groups (MD: 22.1% to 37.5%). During the window ventilation period, the mean indoor/outdoor PM_2.5_ percentage is >60% in five of six groups (except for the three machines with medium-flow setting group: 47.4 ± 15.7%). This finding implies that the efficacy of portable HEPA air cleaner is severely impaired during the period of living room window ventilation.

Multiple linear regressions were performed to evaluate the factors associated with indoor PM_2.5_ levels (Table 4) and indoor/outdoor PM_2.5_ percentage (Table 5). Air cleaner setting (higher airflow speed and machine number), window ventilation, and outdoor PM_2.5_ are significant factors affecting indoor PM_2.5_ and indoor/outdoor PM_2.5_ percentage. For indoor PM_2.5_: R = 0.879 and R square (R^2^) = 0.773; and for indoor/outdoor PM_2.5_ percentage: R = 0.808 and R^2^ = 0.653. The outdoor wind speed, however, did not significantly affect indoor PM_2.5_ and indoor/outdoor PM_2.5_ percentage.

## 4. Discussion

This study showed that HEPA air cleaners can effectively reduce the indoor PM_2.5_ level and achieve good indoor air quality. Under the best air cleaner setting (three machines with medium-airflow setting), the HEPA air cleaner can achieve an indoor PM_2.5_ level of 9.7 µg/m^3^, an indoor/outdoor PM_2.5_ percentage of 22.1%, and PM_2.5_ improvement of up to 56%. However, the efficacy of the HEPA air cleaner is variable due to the different settings of the air cleaner and the environment. Airflow speed, machine number, outdoor PM_2.5_ level, and window ventilation are significant factors affecting indoor PM_2.5_.

There are approximately three-fold differences observed between air cleaners with medium-flow and low-flow settings. According to the coefficient and R square change of each variable in the multiple linear regression model (Table 4 and Table 5), the effect size of 1 machine with medium flow is similar to 2 machines with low flow. The effect size is larger in 2 machines with medium flow than 3 machines with low flow. These findings suggest that the more machines are not always better. Airflow speed, in addition to the machine number, is also an important factor affecting the PM_2.5_ outcomes and HEPA air cleaner efficacy.

The indoor PM_2.5_ level is closely affected by outdoor PM_2.5_, via window ventilation. Previous literature shows good correlations between indoor and outdoor PM_2.5_ (linear regression R = 0.66–0.91) in buildings without air cleaners [25,26], and the average indoor/outdoor ratio ranged from 0.69 to 0.94 [25], which corresponds with the result of our study (when air cleaners are turned off, the average indoor/outdoor ratio = 0.76, linear regression R = 0.794).

Our study clearly shows that window ventilation severely reduces the efficacy of HEPA air cleaners. In Figure S5 (Supplementary Materials), which shows the detail 24-h trends of indoor/outdoor PM_2.5_ percentage in the six study groups, rapid declines in indoor/outdoor PM_2.5_ percentage are observed right after air cleaners turn on at 18:30. However, recurrent high peaks of indoor/outdoor PM_2.5_ percentage are frequently found between 20:00–21:00, which correlates with the time of window ventilation. This implies that during the period of window ventilation, the air volume through the window is large enough to overwhelm the filtration capacity of air cleaners. Therefore, the appropriate limitation of window opening for ventilation is crucial to ensure the efficacy of the HEPA air cleaner. However, window closure would induce an elevation of the indoor CO_2_ level. Our window opening protocol provides an example of the minimal ventilation required to maintain the indoor CO_2_ near upper normal limit.

The current PM_2.5_ air pollution status in Taiwan is better than that in South Asia, North Africa, and the Middle East, but still above the normal range of the air quality index of the United States (AQI, 0–12 µg/m^3^) [27]. Furthermore, the 2021 World Health Organization air quality guideline suggests an annual mean PM_2.5_ level of 5 µg/m^3^, because in recent years, it has been considered that there is no actually safe threshold for PM_2.5_, and therefore people should keep PM_2.5_ concentration as low as possible [28,29]. Our study demonstrates a good example that under the ideal window ventilation condition, adequate machine number, and airflow, the best efficacy of portable HEPA air cleaners can achieve an indoor PM_2.5_ level within the normal AQI range (outdoor 45.6 µg/m^3^ vs. indoor 9.7 µg/m^3^, in 3 air cleaner medium flow group) during the air pollution seasons in Southern Taiwan.

Our study has several limitations. First, it was conducted in a single indoor space. Therefore, our study results may not represent the efficacy of HEPA air cleaners in different environments. Second, the window opening protocol cannot be directly applied to buildings with different indoor areas. However, the monitoring of indoor CO_2_ level is an alternative guide for maintaining minimal required ventilation. Third, we did not evaluate the auto-mode of the air cleaner. Although auto-mode is commonly used in real-life, it is difficult to evaluate the relation between airflow speed and air cleaner efficacy.

## 5. Conclusions

This study shows the effectiveness of the portable HEPA air cleaner on improving indoor PM_2.5_ level. However, the efficacy of the air cleaner is also easily affected by several factors. It has been demonstrated that by controlling these factors: adequate machine number, continuous higher airflow (with acceptable noise), and appropriate limitation of window ventilation by using a simple protocol, HEPA air cleaner can significantly reduce the indoor PM_2.5_ to below 10 µg/m^3^. People spend most of their time in indoor environments. Significantly reducing chronic indoor PM_2.5_ exposure by HEPA air cleaners should be an effective method to improve general health. Future air cleaner studies about machine positions, different window ventilation methods, and different HEPA filter conditions are required for further guidance of air cleaner use.

## Figures and Tables

**Table 1:** Features of HEPA filter air cleaners tested in this study.

Air Cleaner Setting	*n*	Airflow Speed (m/s)	Outlet Area (cm^2^)	Estimated CADR (m^3^/h)	Noise (dB)
Low flow	9	0.53 ± 0.11	207	39.5	31.4 ± 1.5
Medium flow	9	1.47 ± 0.1	207	109.5	35.9 ± 0.6
High flow	9	4.34 ± 0.11	207	323.4	56.5 ± 1.6

CADR = clean air delivery rate, HEPA = High-efficiency particulate air.

**Table 2:** Summary of outdoor and indoor PM_2.5_ outcomes.

Group	Flow (*n*)	Outdoor PM_2.5_ µg/m^3^	MD	Indoor PM_2.5_ µg/m^3^	MD	*p* Value	Indoor /Outdoor PM_2.5_ %	MD	*p* Value
1 air cleaner low flow	Off (350)	44.8 ± 10.2	2.6	32.6 ± 10.9	−3.7	<0.001	72.2 ± 19	−10.4	<0.001
	On (349)	47.4 ± 19.3		28.9 ± 11.4			61.8 ± 16.7		
1 air cleaner medium flow	Off (347)	56.7 ± 15.9	−3.4	44.7 ± 8.2	−23.9	<0.001	82.2 ± 17	−40.5	<0.001
	On (344)	53.3 ± 19.6		20.8 ± 7.8			41.7 ± 17.6		
2 air cleaner low flow	Off (348)	47.9 ± 15.1	0.4	35.7 ± 9.1	−15.4	<0.001	78.1 ± 21.1	−37.5	<0.001
	On (350)	48.3 ± 24.2		20.3 ± 11.9			40.6 ± 13.8		
2 air cleaner medium flow	Off (349)	40.5 ± 6.3	0	25.6 ± 4.6	−13.9	<0.001	63.6 ± 9.2	−35.2	<0.001
	On (348)	40.5 ± 4.8		11.7 ± 6.9			28.4 ± 15.3		
3 air cleaner low flow	Off (350)	31.7 ± 5.1	1.1	26 ± 3	−14.2	<0.001	83.3 ± 11.4	−46.1	<0.001
	On (351)	32.8 ± 5.3		11.8 ± 4			37.2 ± 16.3		
3 air cleaner medium flow	Off (351)	46.7 ± 12.3	−1.1	36.2 ± 9	−26.5	<0.001	78.5 ± 11.3	−56.4	<0.001
	On (349)	45.6 ± 7.7		9.7 ± 4.1			22.1 ± 12		
Overall	Off (2095)	44.7 ± 13.8	−0.1	33.5 ± 10.3	−16.3	<0.001	76.3 ± 16.8	−37.7	<0.001
	On (2091)	44.6 ± 16.8		17.2 ± 10.7			38.6 ± 19.8		

MD = mean difference.

**Table 3:** PM_2.5_ outcomes for living room window ventilation during air cleaner operation.

Group	Living Room Window	N	Indoor/Outdoor PM_2.5_ %	MD	*p* Value
1 air cleaner low flow	close	337	60.7 ± 15.9	30.9	<0.001
	open	12	91.6 ± 10.4		
1 air cleaner medium flow	close	329	40.7 ± 16.9	22.7	<0.001
	open	15	63.4 ± 17.9		
2 air cleaner low flow	close	335	39.7 ± 12.5	22.1	<0.001
	open	15	61.8 ± 22.5		
2 air cleaner medium flow	close	333	26.8 ± 13.2	37.5	<0.001
	open	15	64.3 ± 15		
3 air cleaner low flow	close	334	35.7 ± 14.3	32.3	<0.001
	open	17	68 ± 22.2		
3 air cleaner medium flow	close	334	21 ± 10.5	26.4	<0.001
	open	15	47.4 ± 15.7		
Overall	close	2002	37.5 ± 18.9	27.8	<0.001
	open	89	65.3 ± 21.5		

MD = mean difference.

**Table 4:** Multiple linear regression analysis of the factors associated with indoor PM_2.5_.

Factors	B	Standard Error	Beta	R^2^ Change	*p* Value
(Constant)	12.651	0.510			<0.001
1 machine low flow	−5.811	0.383	−0.121	0.014	<0.001
1 machine medium flow	−16.722	0.409	−0.346	0.067	<0.001
2 machines low flow	−14.905	0.381	−0.311	0.081	<0.001
2 machines medium flow	−19.787	0.376	−0.411	0.089	<0.001
3 machines low flow	−16.276	0.417	−0.340	0.077	<0.001
3 machines medium flow	−24.212	0.389	−0.504	0.132	<0.001
Outdoor PM_2.5_	0.464	0.007	0.537	0.304	<0.001
Window ventilation	6.361	0.471	0.100	0.010	<0.001
Outdoor wind speed	−0.104	0.122	−0.008		0.392

R = 0.879, R^2^ = 0.773, adjusted R^2^ = 0.773.

**Table 5:** Multiple linear regression analysis of factors associated with indoor/outdoor PM_2.5_ percentage.

Factors	B	Standard Error	Beta	R^2^ Change	*p* Value
(Constant)	85.748	1.245			<0.001
1 machine low flow	−13.941	0.934	−0.147	0.022	<0.001
1 machine medium flow	−33.074	0.996	−0.347	0.110	<0.001
2 machines low flow	−35.025	0.929	−0.370	0.094	<0.001
2 machines medium flow	−48.803	0.916	−0.515	0.138	<0.001
3 machines low flow	−41.898	1.016	−0.444	0.096	<0.001
3 machines medium flow	−54.154	0.948	−0.572	0.165	<0.001
Outdoor PM_2.5_	−0.210	0.017	−0.123	0.015	<0.001
Window ventilation	14.087	1.149	0.113	0.013	<0.001
Outdoor wind speed	−0.332	0.296	−0.013		0.263

R = 0.808, R^2^ = 0.653, adjusted R^2^ = 0.652.

## Data Availability

Data are contained within the article or Supplementary Materials.

## Supplementary Materials

The following supporting information can be downloaded at: https://www.mdpi.com/article/10.3390/ijerph191811517/s1, Figure S1. Window ventilation protocol and indoor CO_2_ trend. Figure S2. Correlation of the 2 AirBox PM_2.5_ sensors. Figure S3. Floor plan of the study apartment. Figure S4. Details of the changes in (A) outdoor and (B) indoor PM_2.5_ levels of each study group before and after air cleaner use. Figure S5. Detail trends of indoor/outdoor PM_2.5_ percentage in the six study groups. Figure S6. Box plots of outdoor wind speed distributions in each study groups.
