# Supplementary material for: Efficacy of HEPA Air Cleaner on Improving Indoor Particulate Matter 2.5 Concentration
Source: Int J Environ Res Public Health. 2022 Sep 13;19(18):11517. doi: 10.3390/ijerph191811517 (PMC9516965; doi:10.3390/ijerph191811517)
Supplement: Supplementary file 1 [file ijerph-19-11517-s001.zip › ijerph-1853012-supplementary.pdf]

Supplementary File

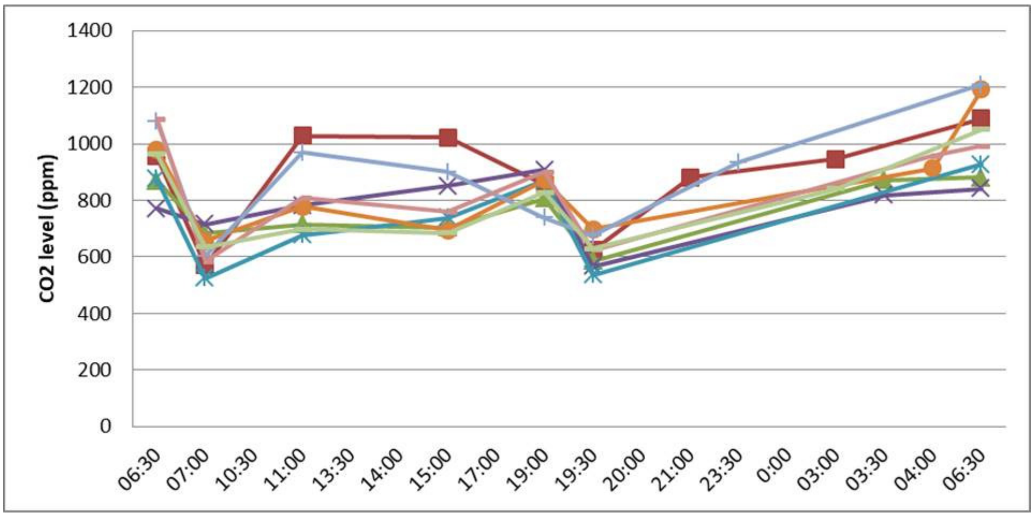

Figure S1. Window ventilation protocol and indoor CO_2 trend. Serial CO_2 was measured for eight separate days using the window ventilation protocol, and results showed marginal CO_2 elevation at the end of the test (mean: 1014 ppm, range: 841–1208 ppm), compared with that at the beginning of the test (mean: 947 ppm, range: 770–1086 ppm, represent the result of the usual window ventilation habits of patients in the previous day: living room window were kept half open in the evening and closed during the day and after sleep. The windows in the kitchen and bathrooms were kept open all day). One adult stayed in the apartment from 6:30 am to 6:30 pm; then, two adults and one child stayed in the apartment from 6:30 pm to 6:30 am.

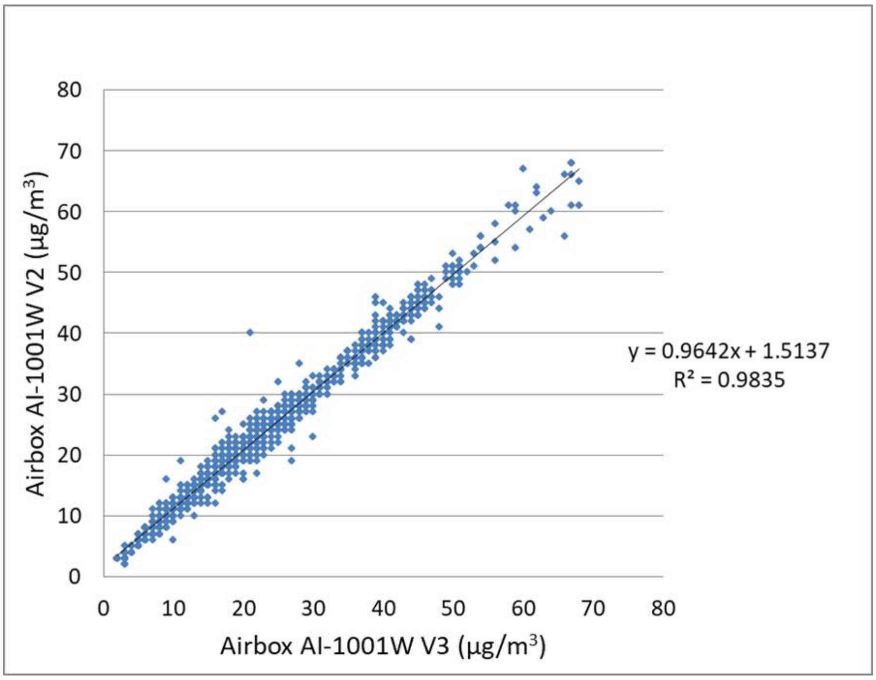

Figure S2. Correlation of the 2 AirBox $PM_{2.5}$ sensors. Linear regression analysis showed very good correlation of the $PM_{2.5}$ data between the two sensors, R = 0.99, R^2 = 0.98.

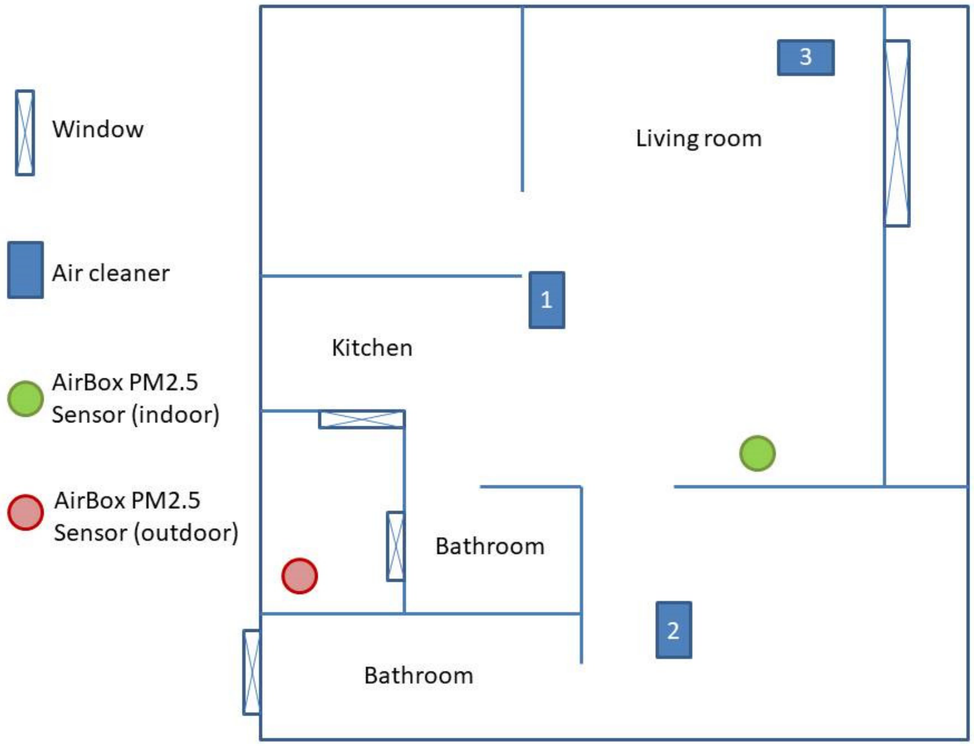

Figure S3. Floor plan of the study apartment. The floor plan of the apartment showed the positions of two AirBox $PM_{2.5}$ sensors (green circle: indoor $PM_{2.5}$ sensor; red circle: outdoor $PM_{2.5}$ sensor) and three air cleaners (blue rectangle).

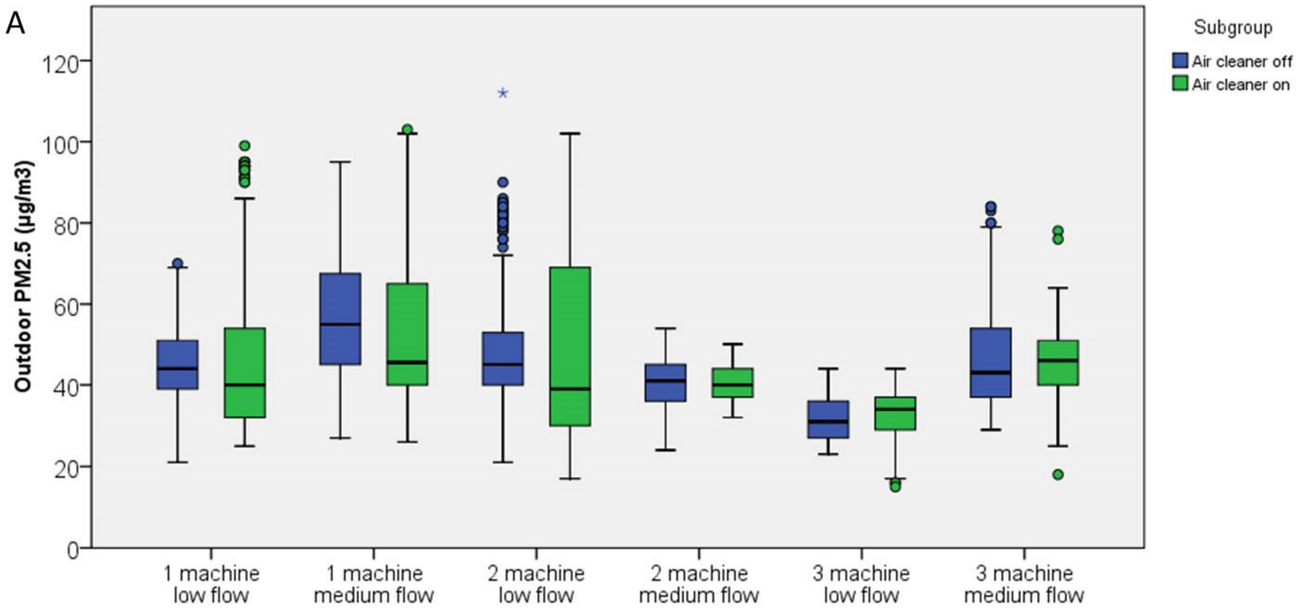

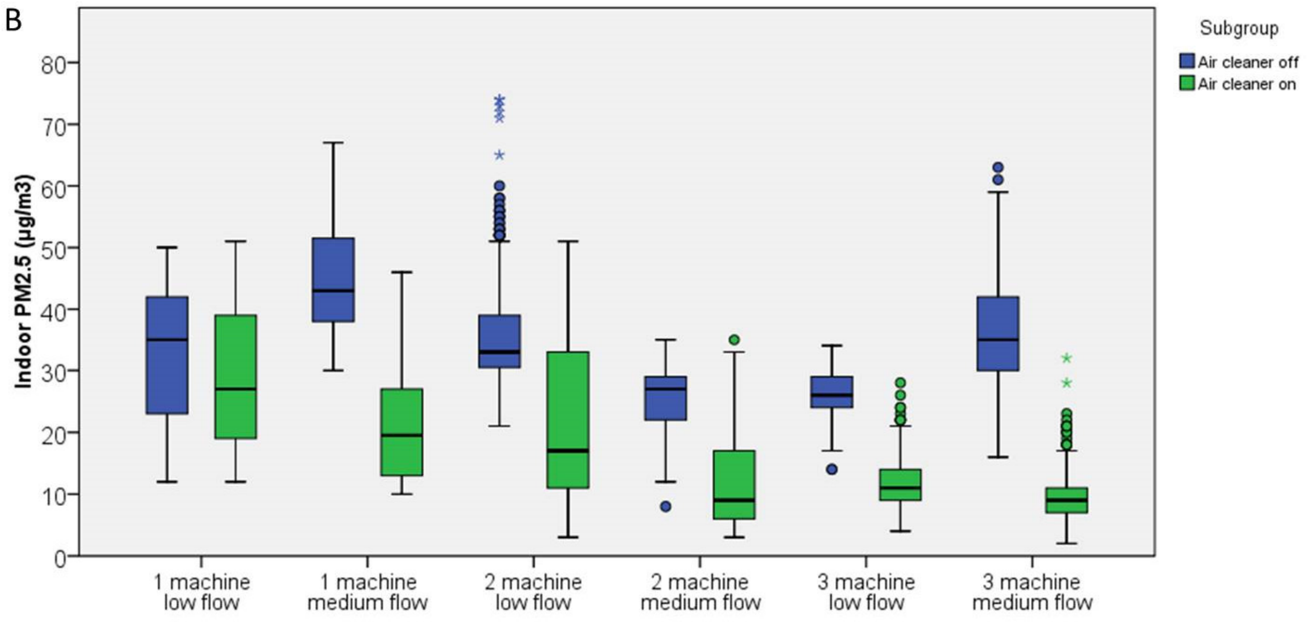

Figure S4. Details of the changes in (**A**) outdoor and (**B**) indoor $PM_{2.5}$ levels of each study group before and after air cleaner use. * outliers.

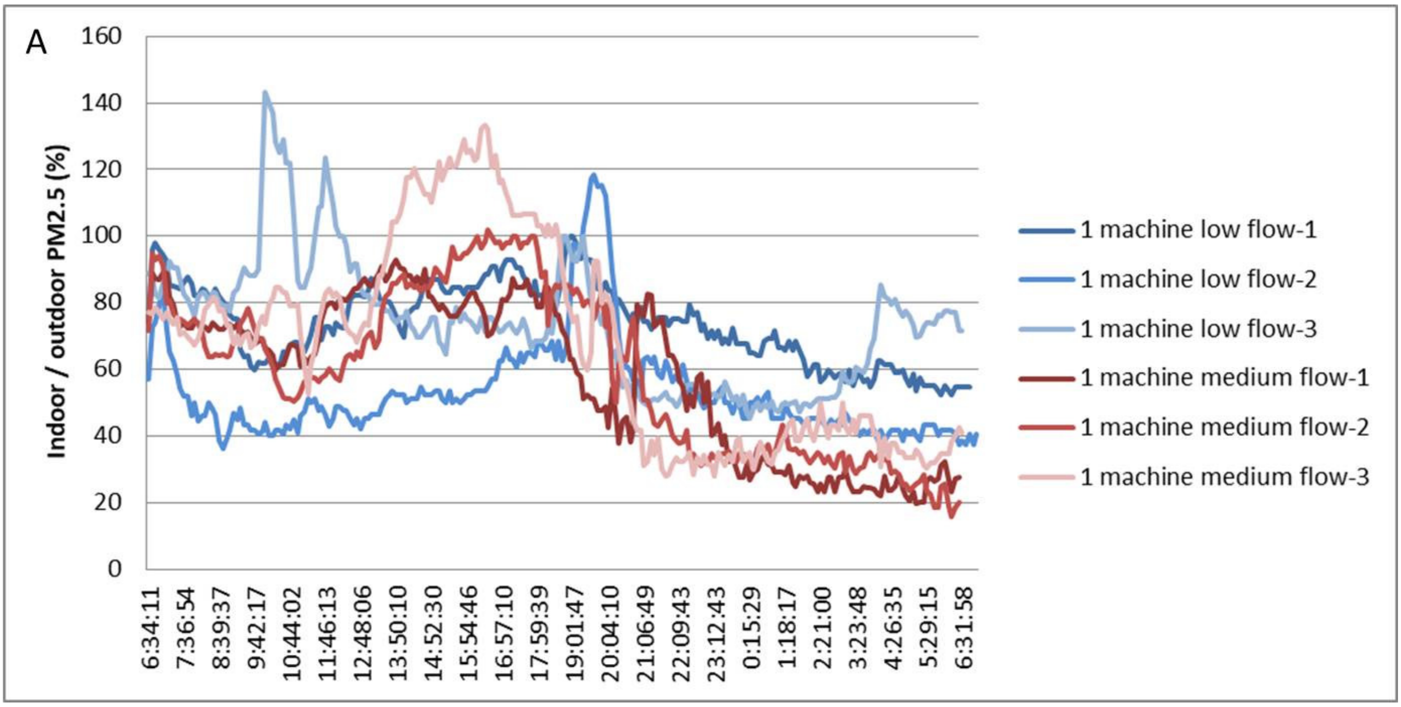

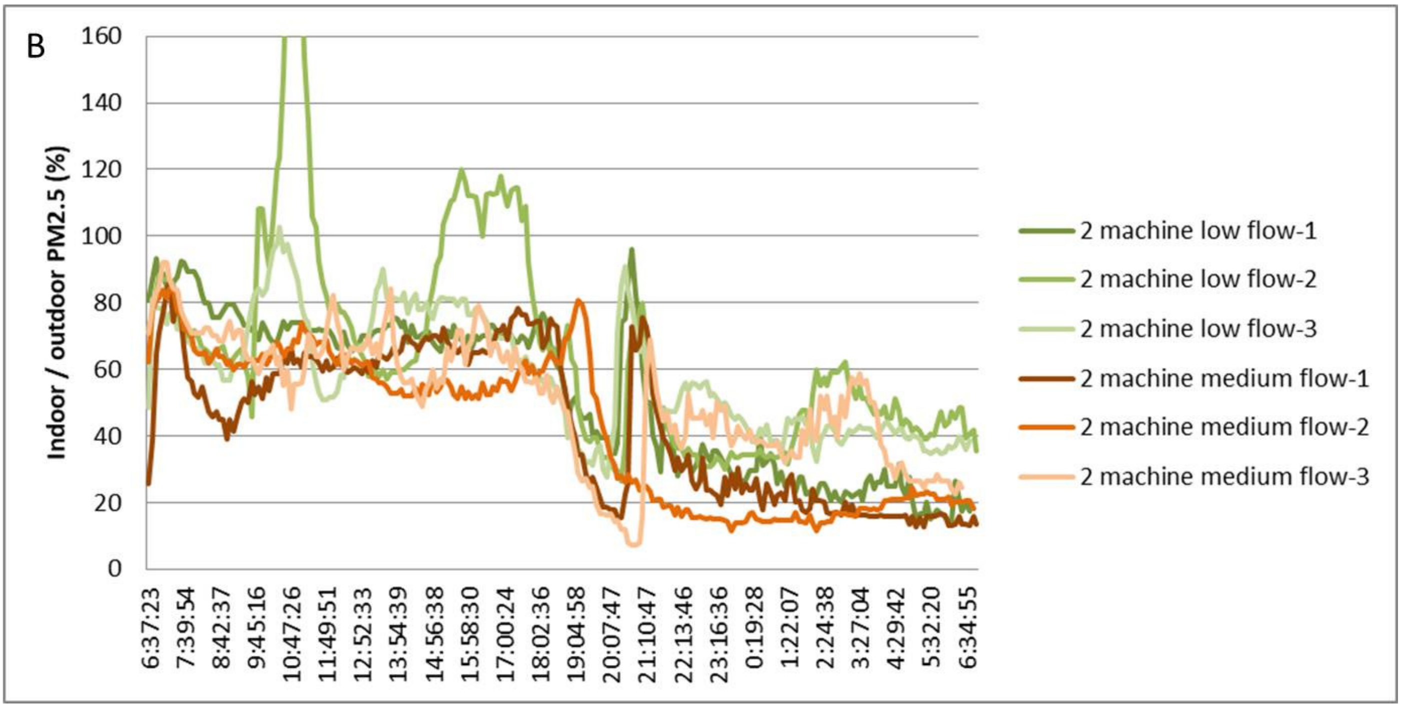

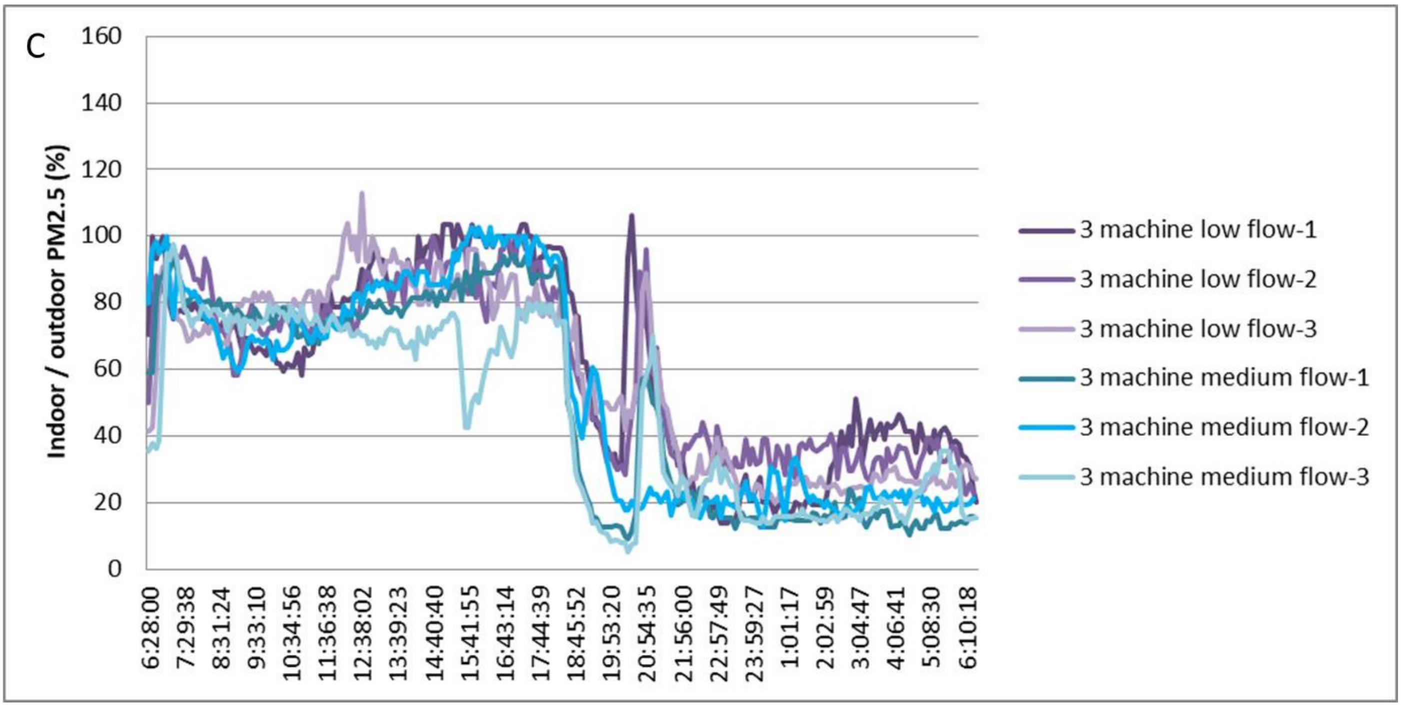

Figure S5. Detail trends of indoor/outdoor $PM_{2.5}$ percentage in the six study groups. (**A**) One machine with low-flow setting and one machine with medium-flow setting groups. (**B**) Two machine with low-flow setting and two machines with medium-flow setting groups. (**C**) Three machines with low-flow setting and three machines with medium-flow setting groups. Air cleaner was closed in the first 12-hour period (6:30–18:30) and then opened in the next 12-hour period (18:30–6:30).

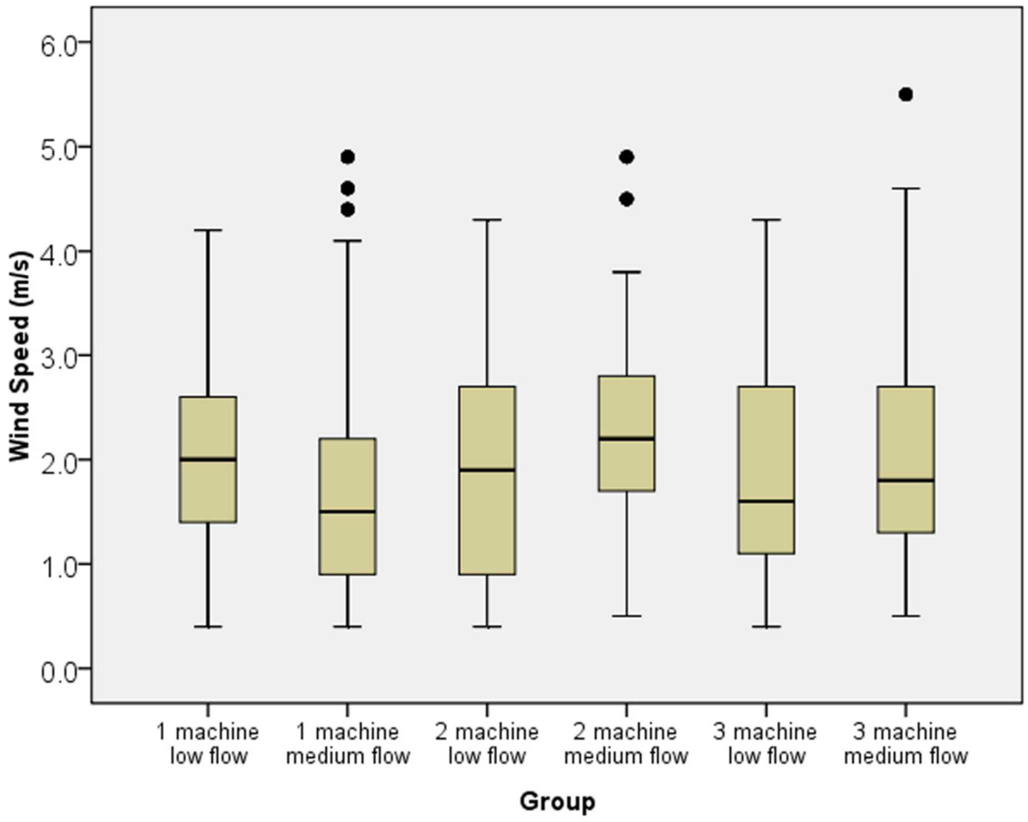

Figure S6. Box plots of outdoor wind speed distributions in each study groups.
